# Soil Salt Distribution and Tomato Response to Saline Water Irrigation under Straw Mulching

**DOI:** 10.1371/journal.pone.0165985

**Published:** 2016-11-02

**Authors:** Yaming Zhai, Qian Yang, Yunyu Wu

**Affiliations:** 1 Key Laboratory of Efficient Irrigation-Drainage and Agricultural Soil-Water Environment in Southern China, Hohai University, Nanjing, 210098, China; 2 College of Water Conservancy and Hydropower, Hohai University, Nanjing 210098, China; 3 Henan Vocational College of Agriculture, Department of Gardening, Zhengzhou, China; University of Vigo, SPAIN

## Abstract

To investigate better saline water irrigation scheme for tomatoes that scheduling with the compromise among yield (*Y*_*t*_), quality, irrigation water use efficiency (*IWUE*) and soil salt residual, an experiment with three irrigation quotas and three salinities of irrigation water was conducted under straw mulching in northern China. The irrigation quota levels were 280 mm (W1), 320 mm (W2) and 360 mm (W3), and the salinity levels were 1.0 dS/m (F), 3.0 dS/m (S1) and 5.0 dS/m (S2). Compared to freshwater, saline water irrigations decreased the maximum leaf area index (*LAI*_*m*_) of tomatoes, and the *LAI*_*m*_ presented a decline tendency with higher salinity and lower irrigation quota. The best overall quality of tomato was obtained by S2W1, with the comprehensive quality index of 3.61. A higher salinity and lower irrigation quota resulted in a decrease of individual fruit weight and an increase of the blossom-end rot incidence, finally led to a reduction in the tomato *Y*_*t*_ and marketable yield (*Y*_*m*_). After one growth season of tomato, the mass fraction of soil salt in plough layer under S2W1 treatment was the highest, and which presented a decline trend with an increasing irrigation quota. Moreover, compared to W1, soil salts had a tendency to move to the deeper soil layer when using W2 and W3 irrigation quota. According to the calculation results of projection pursuit model, S1W3 was the optimal treatment that possessed the best comprehensive benefit (tomato overall quality, *Y*_*t*_, *Y*_*m*_, *IWUE* and soil salt residual), and was recommended as the saline water irrigation scheme for tomatoes in northern China.

## Introduction

Agricultural waters account for 95% of the total water consumption in the world [1]. For China, particularly those irrigation areas of northern China, inadequate rainfall and limited surface water supply have seriously impeded the development of agriculture [2]. Since 1992, the irrigation areas in northern China suffered severe drought, leading to a great loss of grain yields that was approximate 17 500 million kilogram every year. Because of the water shortages, some areas in northern China maintained the development of agriculture by excessively exploiting the underground water, and this resulted in a 500 000 km^2^ ground falls in the border regions of Beijing, Tianjing and Hebei province [3–4]. Presently, China has applied various technologies to deal with the water shortage problems in the northern irrigation areas, including the water conservancy engineering [5–6], the biological water-saving technology [7–8], the optimize arrangement of crops [9], the reclamation of sewage [10] and the saline water irrigation technology [11–12].

Although irrigation with saline water relieves fresh water resource shortages in varying degrees, the improper use of saline water (such as the use of saline water with excessive salinity or insufficient irrigation with saline waters) may result in combinations of water and saline stress that lead to the secondary salinization and a series of environmental problems [13–14]. Therefore, the dynamic and distribution of soil salts under saline water irrigation were extensively studied [2, 13, 15–16]. The water-salt dynamic models were used to understand the distribution of soil salts under saline water irrigation, thus providing a reference for the decision of saline water irrigation scheme [13].

Tomatoes are sensitive to salts that they can not survive under high salinity condition or only survive with decreased yields [17]. To alleviate the deleterious effects of the salt, several methods such as mixed irrigation with freshwater and saline water [18], and rotated irrigation with freshwater and saline water [19] have been applied. Some studies have specifically examined the effects of saline water irrigation on tomato growth, development, quality, yield and blossom-end rot incidence (*BER*_*i*_) [20–23]. Tomato organoleptic parameters, such as soluble solids, fructose, glucose, titratable acid, and amino acid contents, increase with increasing salinity [20, 24–28]. Of these factors, suitable salt stress can be applied to improve the fruit quality. Although positive indicators of tomato quality have been obtained under saline conditions, it has been reported that tomato yield is negatively affected by increasing salinity [17, 29]. Furthermore, although the tomato *BER*_*i*_ is commonly considered as a physiological disorder that caused by calcium deficiency [30], salt stress is one of the main environmental factors to aggravate *BER*_*i*_ [31–32].

On the other hand, water is also an important factor that affects the growth and development of tomatoes [33]. A reasonable irrigation quota is beneficial for the tomatoes to obtain high yield and good quality. Study on the tomato deficit irrigation has shown that the soluble solid, vitamin C, sugar, acid, and sugar to acid ratio in the fruits increase with a lower water supply, resulting in an improved overall quality of tomatoes [34–35]. Moreover, under deficit irrigation condition, the acid invertase, neutral invertase, proline, glucose and fructose in the tomato fruits are increased, and this helps the tomato plants to be more adaptive to the drought stress [36–37]. However, low water supply reduces the tomato yields, which decreases the weight of single fruit but has no significant effects on the fruit number [33, 38]. On the contrary, excessive water supply leads to the spindling of tomatoes, limits the tomato physiological and reproductive growth, finally causes a low tomato yield [39–40]. In addition, water supply has certain connections with the tomato *BER*_*i*_, while water whether to directly affect the *BER*_*i*_ is still not fully understood [41]. Besides, under the condition of irrigation combined with mulching, soil salt was found to move towards the edge of the mulch, thus the salinity within the plant root-zone was decreased, which created a suitable environment for the growth of tomato [42].

Presently, there are some studies focused on the regularity of soil salt distribution under the saline water irrigation, but which under special control conditions such as straw mulching have been less studied. Besides, although many studies have independently investigated the tomato responses to different salinity of irrigation waters and different irrigation amount, few studies have looked into their combined effects on the tomato growth, quality, yield and *BER*_*i*_. And most of all, in southern China, it is important for the irrigation agriculture to find a tomato irrigation scheme that not only maintains normal output but also with a better fruit quality, a higher *IWUE* and a relatively lower salt residual in plough layer. In this experiment, the tomatoes were treated with different irrigation water salinities and irrigation quotas under a mulch of dry straws, and the tomato responses and the soil salt distribution under these treatments were compared and analyzed. For crop performance, we hypothesized that all environmental factors have the same effects on tomato growth and development. The objectives of this study were to understand the soil salt distribution laws and the tomato responses to different saline water treatments, and to find out improved saline water irrigation methods with best comprehensive effects that improve the fruit quality, increase the tomato *IWUE* and relatively reduce the soil salt residual but do not significantly decrease the tomato yields in northern China. These treatments were compared with a freshwater (F) irrigation treatment.

## Materials and Methods

### Experiment Conditions

The experiment was carried out in 2014 (May-September) at Modern Agriculture Park of Xinzhou city, Shanxi Province, northern China (The experiment was permitted by the owner of the land named Dong Qiuyue). The experimental site belongs to a temperate continental monsoon climate and enjoys four clear seasons. The annual mean temperature of the experimental site is 4.3°C-9.2°C and the mean precipitation is 345 mm-588 mm (data from 1960–2010). In addition, heavy rains in the experimental site have the characters of small area, short duration, strong intensity and unevenness in time and space, they mainly happened during July-August, accounting for 83.7% of the total rainy days. The soil type in the experimental fields is sandy loam, with bulk density of 1.34 g/cm^3^, organic matter of 1.36%, salt content of 1.13 g/kg, alkali-hydrolyzale N of 94.58 mg/kg, available P of 18.44 mg/kg, and available K of 77.86 mg/kg, in 0–20 cm soil layer.

### Experimental Design

The experiment was conducted in a plastic-covered greenhouse, and the tomato type for experiment was “Yinshidahong”. After the soils were ploughed uniformly, the seedlings were transplanted to the experimental blocks. During the seedling stage, the same field managements were applied among different treatments. Soil ridges were constructed for tomatoes, and the ridges were 4.4 m length and 0.6 m width, with a 1.4 m distance between them. Two lines of tomatoes were transplanted to one ridge with the line spacing of 0.3 m and the row spacing of 0.4 m, and the planting density was about 3.6×10^4^ plants/hm^2^. To provided the nutrients necessary for tomato plant growth, the experiment fields were fertilized with 700 kg/hm^2^ of N: P_2_O_5_: K_2_O = 1:2:2 compound fertilizer. The 3–5 cm dry straws of paddy rice were used as the mulch material, of which the mulch amount was 4000 kg/hm^2^, mulching uniformly at 20 days after seedling transplanting.

According to the experience from early study [43], we designed three saline water treatments: 1.0 dS/m (F), 3.0 dS/m (S1), 5.0 dS/m (S2), combined with three irrigation quotas: 280 mm (W1), 320 mm (W2), 360 mm (W3), grouped as 9 treatments, each treatment was replicated 3 times, as shown in Table 1. Three ridges of tomatoes were gathered as one treatment, which was applied with the same salinity and irrigation quota. Flood irrigation according to the local practice was applied. For the treatments with different irrigation quotas, the irrigation times was the same (13 times), while the irrigation amount each irrigation time was different. An impermeable membrane at a depth of 60 cm was used between the different treatments to prevent lateral seepage of the irrigation water. In this experiment, the freshwater (F) was the local underground water with the EC of 1.0 dS/m, saline water (S) were prepared with these underground waters. Details regarding the saline water were presented in Table 2.

**Table 1:** Experimental design.

Treatment	FW1	FW2	FW3	S1W1	S1W2	S1W3	S2W1	S2W2	S2W3
EC (dS/m)	1.0	1.0	1.0	3.0	3.0	3.0	5.0	5.0	5.0
Irrigation quota(mm)	280	320	360	280	320	360	280	320	360

**Table 2:** The ionic compositions of the different irrigation water treatments.

EC (dS/m)	Ionic content (mmol/L)							
	Na^+^	K^+^	Mg^2+^	Ca^2+^	CO_3_^2-^	HCO_3_^-^	Cl^-^	SO_4_^2-^
1.0	2.6	0.9	3.8	0.5	0.4	5.8	2.4	1.4
3.0	13.2	3.7	7.3	2.0	0.4	12.9	9.5	5.4
5.0	25.9	5.9	10.2	2.7	0.4	22.3	16.2	9.1

The lateral tomato plant branches were removed during the growth period, and topping treatments were applied in a timely manner. Each tomato plant was allowed to reserve 4 fruit sequences. Pest control was conducted according to the actual situation in the experimental fields.

### Measurements

The concentrations of K^+^ and Na^+^ in the saline water were measured using flame photometry method. The concentrations of Ca^2+^ and Mg^2+^ were measured using the atomic absorption spectrophotometry method. The concentrations of Cl^-^, SO_4_^2-^ were measured using the anionic chromatography method. The concentrations of CO_3_^2-^ and HCO_3_^-^ were measured using the double indicators-neutralization titration method [44].

The leaf area index (*LAI*) was measured at every stage using an LAI 2000 Plant Canopy Analyzer (Li-Cor Biosciences USA). The maximum *LAI* (*LAI*_*m*_) of each treatment was extracted for analysis.

The tomato yield (*Y*_*t*_), *BER*_*i*_ and marketable tomato yield (*Y*_*m*_) were determined at the harvest stage, and tomato fruits were picked manually every 3–5 days. For each harvest, the number and weights of good fruits and the fruits with *BER*_*i*_ were recorded. *Y*_*m*_ was calculated as follows (The malformed fruits were very little and ignored here) [29]:

$$Y_{m}=Y_{t}\left(1\text{-}BER_{i}\right)$$

To determine the tomato quality in each treatment, 20 tomato fruits with red or orange colors were collected randomly to measure the quality indexes. The quality indexes included the volume (*V*_*F*_), density (*ρ*_*F*_), soluble solids content (*D*_*s*_), total acidity content (*G*), vitamin C content (*V*_*C*_) and sugar/acid ratio (*RSA*). The *V*_*F*_ was measured using the displacement method, and the *ρ*_*F*_ was calculated based on the tomato volume and weight. In addition, *D*_*s*_ was measured using an ACT-1E digital refractometer (ATAGO Company, Japan), and the total sugar content was measured using the Fehling reagent titration method. The *G* was measured using the sodium hydroxide titration method, and the *V*_*c*_ content was measured using the 2, 6-dichloroindophenol titrimetric method [17, 45].

*IWUE* (kg/m^3^) was calculated as [46]:

IWUE=Y/I

Where, *I* was the irrigation amount (m^3^) during the whole growth stage of tomatoes.

The soil samples for measuring the mass fraction of salt in the soil profile (0–20 cm, 20–40 cm, 40–60 cm, 60–80 cm) was collected on September 18^th^, after the last harvest of tomatoes.

### Data Analysis

The data were statistically compared using a one-way ANOVA with Duncan’s Multiple Range Test at the 0.05 probability level (using the SPSS software, Version 17.0) [47]. The quality indexes for the Principal Component Analysis (PCA) were also obtained from the SPSS software. The principal components of the quality indexes were extracted following the principle of “eigenvalue > 1, cumulative contribution rate > 80%” [48].

### Projection pursuit (PP) Model

The PP model is a well-developed method for selecting the optimal scheme when there were various schemes with various evaluation indexes. Here, the PP model is used to select the optimal irrigation scheme from the 9 treatments of this experiment, and the optimal irrigation scheme should possess the best integrated benefits based on the evaluation indexes including the tomato comprehensive quality, *Y*_*t*_, *Y*_*m*_, *IWUE* and the soil salinity after irrigation.

The essence of PP model is to use computer technology to project high dimensional data to lower dimensional space, and search for the projection which could well reflect the characters of high dimensional data, then study the structures of high dimensional data in a low dimensional space. The modeling method is as follows [49–50]:

1. Establish the evaluation matrix. Suppose the number of treatments is *n*, number of evaluation indexes is *p*, the *j*^*th*^ index of *i*^*th*^ sample is *x*_ij_*, then the evaluation indexes could be expressed by an *n×p* matrix X*.
2. Quantify the evaluation indexes. In order to eliminate the differences of dimension, following measures are taken:
  For the "the larger the better" index:
  xij=xij*-min(x*j)max(xj*)-min(xj*)
  For the "the smaller the better" index:
  $$x_{ij}=\frac{\text{max}(x_{j}{}^{*}){-x}_{ij}{}^{*}}{\text{max}(x_{j}{}^{*})-\text{min}(x_{j}{}^{*})}$$
  A new *n×p* matrix *X* can be obtained based on the qualified indexes.
3. Linear projection. The essence of linear projection is to observe the data from different angles, to search for the best projective direction which could well reflect the characters of the data, therefore, suppose the unit vector *a =* {*a*_*1*_,*a*_*2*_,*…a*_*p*_} as the one dimensional projective direction, and *z*_*i*_ as the one dimensional projective eigenvalue.
  $$z_{i}=\sum_{j=1}^{p}a_{j}\cdot x_{ij}\;(i=1,\;2,\;3\cdot \cdot \cdot ,\;n;\;j=1,\;2,\;3\cdot \cdot \cdot ,\;p)$$
4. Constructs an object function for projection. Express the object function (*Q*_*(a)*_) as the product of distances between classes and density between classes:
  $$Q_{\left(a\right)}=S_{z}\cdot D_{z}^{}$$
  Where *S*_*z*_ is the standard value of projective eigenvalue *z*_*i*_, also named distances between classes, *D*_*z*_ is the density between classes of *z*_*i*_.
  $$S_{z}=\sqrt{\frac{\sum_{i=1}^{n}{\left(z_{i}\text{-}E\left(z\right)\right)}^{2}}{n\text{-}1}}$$
  Where *E*_*(z)*_ is the average of the array {*z*_*i*_ *|i =* 1~*n|*}.
  $$D_{Z}=\sum_{i=1}^{n}\sum_{k=1}^{n}\left(R\text{-}r_{ik}\right)\cdot f\left(R\text{-}r_{ik}\right)$$
  Where, *R* is window radius of local density;
  $$r_{ik}=\left|r_{i}{\text{-}r}_{k}\right|$$
  $$f\left(t\right)=\{\begin{array}{c} 0\;t\geq 0\\ 1\;t\text{<}0 \end{array}$$
  i, k=1, 2, 3⋅⋅⋅n.
5. Optimize the object function by maximization:
  $$\text{max}Q_{\left(a\right)}=S_{z}\cdot D_{z}^{}$$
  $$s.t.\sum_{j=1}^{p}a^{2}\left(j\right)=1,$$
  |a(j)|≤1
6. Evaluation. The contribution of evaluation index can be obtained according to the best projective direction, and the stand or fall of the treatments can be also obtained based on the *z*_*i*_ value.

In this study, the PP model was built using the Matlab software (Version 7.1), and the Real Adaptive Parallel Genetic Algorithm (*RAGA*) was used to optimize the PP model. Before optimizations, the main parameters were set as: the original population size *n* = 400, the probabilities of crossover *P*_*c*_ = 0.8, the probabilities of mutation *P*_*m*_ = 0.8, the number of excellent individuals *N*_*e*_ = 20, *α* = 0.05 and accelerating times *N*_*a*_ = 20 [51].

## Results

### Effects of different treatments on the *LAI*_*m*_ of tomato

**Fig 1:**
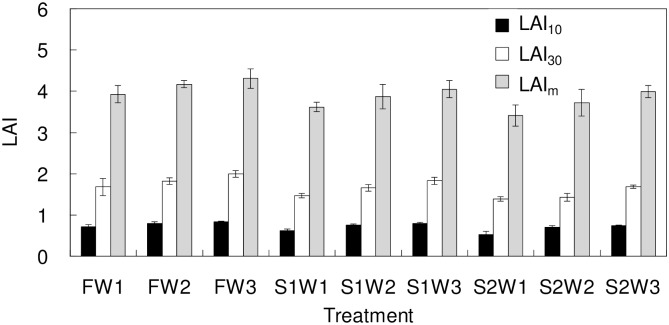
Effects of different treatments on the leaf area index (*LAI*) (W1, W2 and W3 represent the three irrigation quotas of 280, 320 and 360 mm respectively. F, S1 and S2 represent the three water salinities of 1.0, 3.0 and 5.0 dS/m. Each value is the mean ± SD (n = 3)). *LAI*_*10*_ and *LAI*_*30*_ represent the *LAI* at 10 and 30 days after transplant, corresponding to the seedling stage and the flowering stage, respectively. *LAI*_*m*_ represent the maximum *LAI* during the whole growth stage of tomato.

Fig 1 shows the effects of different saline water treatments on the *LAI* of tomatoes. The *LAI* at 10 days after transplant were in a range of 0.53–0.84, and *LAI* at 30 days after transplant ranged from 1.39 to 1.99. Under the same irrigation quota, the tomato *LAI*_*m*_ decreased as the increase of salinity. *LAI*_*m*_ under W1 irrigation quota presented most dramatic decrease, which of S2 was 13.2% lower than that of F, indicating that low irrigation quota combined with high salinity limited the increase of tomato leaf area. On the other hand, under the three salinity levels, the tomato *LAI*_*m*_ all increased as the increase of irrigation quota, of which 5 dS/m increased the tomato *LAI*_*m*_ most significantly that was 17.0% compared between W1 and W3. This probably due to the salt leaching effects of high irrigation quota that relieved the salt stress for the tomato growth and development.

### Effects of different treatments on tomato quality

**Fig 2:**
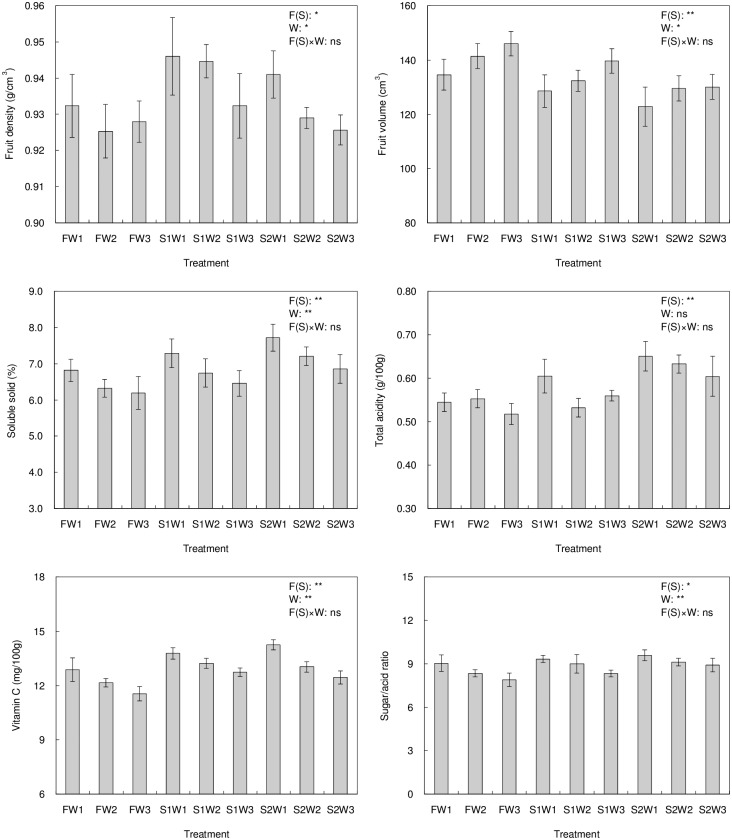
The effect of saline water treatments on main quality indicators of tomato (W1, W2 and W3 represent the three irrigation quotas of 280, 320 and 360 mm respectively. F, S1 and S2 represent the three water salinities of 1.0, 3.0 and 5.0 dS/m. The quality index values are the means of three replications. Each value is the mean ± SD (n = 3). * and ** represent that the treatment has significant (*p*<0.05) and much significant (*p*<0.01) effects on the value of quality index).

Fig 2 gives the values of quality indexes with different irrigation treatments. Overall, lower irrigation quota or higher salinity increased the *ρ*_*F*_, *D*_*S*_, *G*, *V*_*C*_ and *RSA* but decreased the *V*_*F*_ of tomatoes. From the effects of irrigation quota and salinity and their combinations on the tomato quality, it was concluded that the salinity of irrigation waters significantly affected the *ρ*_*F*_, *V*_*F*_, *D*_*S*_, *G*, *V*_*C*_ and *RSA* of tomatoes, and the irrigation quota significantly affected the *ρ*_*F*_, *V*_*F*_, *D*_*S*_, *V*_*C*_ and *RSA* of tomatoes, but their combined effects had no significant effects on these quality indexes of tomatoes.

**Fig 3:**
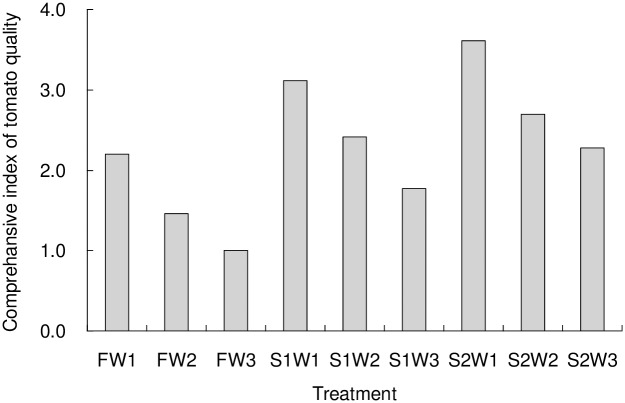
Effects of different treatments on the comprehensive index of tomato quality according to the calculations of principal component analysis (W1, W2 and W3 represent the three irrigation quotas of 280, 320 and 360 mm respectively, F, S1 and S2 represent the three water salinities of 1.0, 3.0 and 5.0 dS/m).

The PCA model was used to extract the principal components of the tomato quality indexes, and the comprehensive indexes of tomato quality with different irrigation treatments were shown as Fig 3. The calculated eigenvalue and accumulating contribution rate was 4.861 and 81.01%, respectively, which retained great original information of tomato quality indexes. A higher comprehensive quality index indicates a higher comprehensive quality (the 6 indexes observed) of the tomatoes. Therefore, in this study, the irrigation treatment with lowest irrigation quota but highest salinity (S2W1) resulted in an overall better tomato quality, comprehensive quality index reaching 3.61; followed by S1W1, with the comprehensive quality index of 3.12; FW3 obtained the most unsatisfactory overall quality of tomatoes, the comprehensive quality index of which was only 1.00. This indicates that decreased the irrigation quota or increased the salinity of irrigation waters resulted in an overall better tomato quality.

### Effects of different treatments on *Y*_*t*_ and *Y*_*m*_

Table 3 shows the effects of different irrigation treatments on the tomato *Y*_*t*_ and *Y*_*m*_. Under the same irrigation quota, the total tomato *Y*_*t*_ and *Y*_*m*_ with S1 salinity had not presented significant (P>0.05) decline trend compared with F salinity, however, the tomato *Y*_*t*_ and *Y*_*m*_ with S2 salinity were significantly (P≤0.05) lower that that with F salinity (by 17.91–20.48% and 22.89–24.76%, respectively). Under the same salinity of irrigation waters, the tomato *Y*_*t*_ and *Y*_*m*_ overall increased with higher irrigation quota. For the single factor of tomato yield, FW3 was proved to be the better treatments that the tomato *Y*_*t*_ and *Y*_*m*_ of which reached 125.8 t/hm^2^ and 120.4 t/hm^2^. Besides, it was found that the *BER*_*i*_ obviously increased under S2 salinity, and was by 9.48–11.74% higher compared with F, which finally led to a negative effect on the tomato *Y*_*m*_ of S2.

**Table 3:** The effect of saline water treatments on total yield (*Y*_*t*_), marketable yield (*Y*_*m*_) and blossom-end rot incidence (*BER*_*i*_) of tomatoes in the two separated harvest month.

Treatment	50–80 DAT			80–110 DAT			Total		
	*Y*_*t*_ (t/ha)	*Y*_*m*_ (t/ha)	*BER*_*i*_ (%)	*Y*_*t*_ (t/ha)	*Y*_*m*_ (t/ha)	*BER*_*i*_ (%)	*Y*_*t*_ (t/ha)	*Y*_*m*_ (t/ha)	*BER*_*i*_ (%)
FW1	44.2bc	41.6c	5.8	64.7ab	60.6bc	6.3	108.9cd	102.3cd	6.1
FW2	50.3b	47.7b	5.2	61.1bc	58.0c	5.1	111.4bc	105.7bc	5.2
FW3	58.1a	55.3a	4.8	67.7a	65.1a	3.9	125.8a	120.4a	4.3
S1W1	40.2cd	37.5cd	6.8	61.0bc	56.4c	7.5	101.2de	93.9de	7.2
S1W2	44.6bc	42.0c	5.9	54.4cd	51.0cd	6.2	99.0def	93.0de	6.0
S1W3	53.3ab	50.4ab	5.5	66.3a	63.0ab	5.0	119.6ab	113.3ab	5.2
S2W1	38.4d	34.8d	9.4	51.0d	44.1de	13.6	89.4f	78.9g	11.8
S2W2	46.8bc	42.7c	8.8	44.2e	39.4e	10.9	91.0ef	82.1fg	9.8
S2W3	45.7bc	41.5c	9.2	54.4cd	49.1cd	9.7	100.1de	90.6ef	9.5

Note: W1, W2 and W3 represent the three irrigation quotas of 280, 320 and 360 mm respectively, F, S1 and S2 represent the three water salinities of 1.0, 3.0 and 5.0 dS/m. The values of *Y*_*t*_ and *Y*_*m*_ are the means of three replications. For *Y*_*t*_ or *Y*_*m*_, means followed by the same letters (a, b, c, d, e, f) are not significantly different at the 5% level according to Duncan’s Multiple Range Test. DAT represent days after transplant.

### The soil salt distribution

**Fig 4:**
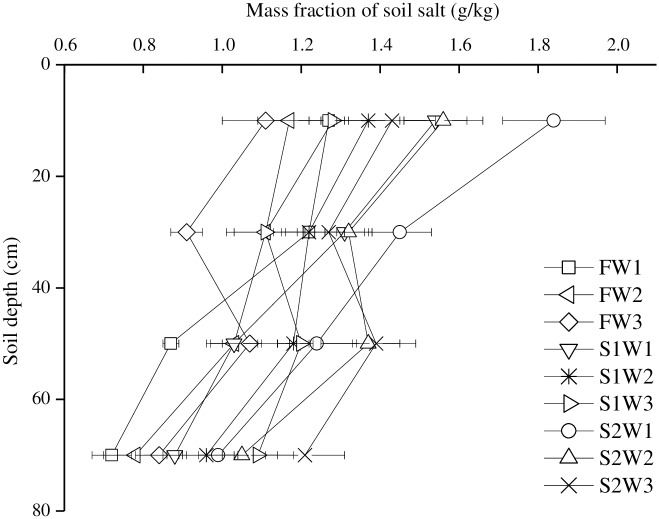
The salt distribution of soil profile after the tomato harvest (W1, W2 and W3 represent the three irrigation quotas of 280, 320 and 360 mm respectively, F, S1 and S2 represent the three water salinities of 1.0, 3.0 and 5.0 dS/m. Each value is the mean ± SD (n = 3)).

Fig 4 showed the salt distribution in soil profile. After a growth season of tomatoes, the soil salts mainly accumulated in the plough layer, mass fraction of which in 0–20 cm, 20–40 cm, 40–60 cm and 60–80 cm layer was 1.11–1.84 g/kg, 0.91–1.45 g/kg, 0.87–1.39 g/kg and 0.72–1.21 g/kg. Under the same irrigation quota, the soil salt mass fraction increased as the salinity of irrigation water increased, of which S2 was 28.83–44.88%, 18.85–39.56%, 29.91–42.53% and 34.61%-44.04% higher compared to F, respectively in the four layers. On the other hand, under the same salinity of irrigation water, salts were presented to move to the deeper soil layer when with higher irrigation quota. W2 and W3 irrigation quota decreased the soil salts in 0–20 cm and 20–40 cm layer compared to W1, but which significantly increased the salts in 40–60 cm and 60–80 cm layer, this indicated that a higher irrigation quota was more effective to leach the salts in plough layer and reduce their accumulation.

### Optimal selection of saline water irrigation treatments

The comprehensive quality, *Y*, *Y*_*m*_, *IWUE* (27.80 kg/m^3^-38.89 kg/m^3^) and soil salt content of plough layer (0–20 cm) were served as the evaluation indexes for the comprehensive benefit assessment of the irrigation treatments. The maximal projective index value that calculated by PP model was 0.2793, the best projective direction *a*_(j)_* = (0.0105, 0.6211, 0.5597, 0.1548, 0.5262), and the projective value of S1W1, S1W2, S1W3, S2W1, S2W2 and S2W3 were ordered to be *z*_(i)_* = (0.9311, 0.9311, 1.8075, 0.0871, 0.3671, 0.7981). A higher projective value indicates a better comprehensive benefit of saline water irrigation treatment. Therefore, S1W3 is the optimal treatment, followed by S1W1 and S1W2.

For S1W3, *Y* and *Y*_*m*_ had not decreased significantly as the salinity of irrigation water increased, and were both the highest among the six saline water irrigation treatments. Besides, although the comprehensive quality index and *IWUE* of S1W3 were not in a superior level relative to other treatments, the index weight (projective direction) of comprehensive quality index (0.0105) was the lowest, thus did not significantly changed the evaluation results. According to the calculations of PP model, 3.0 dS/m salinity of irrigation waters combined with 360 mm irrigation quota was recommended as the best saline water irrigation scheme for the tomato in northern China.

## Discussion

Although the water resources in the world are abundant, the available water resources are insufficient. The total amount of water resources in the world is 1 400 million km^3^, of which the freshwater resources only accounted for 2.8%, and the surface water and shallow ground water that are available accounted for 0.35% of the freshwaters [52]. Agriculture needs a large amount of water and is facing more shortage than other sectors. Presently, 80% of the world’s irrigation system adopts the diversion irrigating method, and the irrigated agriculture will continue to play an important role in meeting the needs of the world population for food [53]. In China, severe droughts often happen in the northern irrigated areas, which limit the sustainable development of agriculture. As a practical method for saving freshwater resources, saline water irrigation in northern China gradually become a hot topic, but until now there were no accepted criterions for saline water management. Thus, it is important to develop suitable management methods for using saline water to meet the challenges of sustainable irrigated agriculture that conserve water resources and have minimum impacts on the soil environment and the crop growth and development.

Our study demonstrated that the lower irrigation quota and higher salinity increased the *ρ*_*F*_, *D*_*S*_, *G*, *V*_*C*_ and *RSA* but decreased the *LAI*_*m*_ and *V*_*F*_ of tomatoes, but there were no significant combining effects on the quality indexes. This was probably because that the tomatoes adjusted initiatively to adapt the water stress when under a lower irrigation quota, the content of osmoregulation substances such as proline, glucose and fructose in tomatoes increased, which had positive effects on the tomato quality [54]. High salinity of irrigation water increased the sugar concentration might due to the enhanced activity of sucrose invertase [55]. A similar study conducted by Beckles [56] also showed that increasing the soil electrical conductivity (EC), either by applying a high ionic solution or by restricting watering, resulted in a higher sugar concentration per fruit.

Early study showed that when the insufficient water supply limited the vegetative growth of tomatoes, the fruits would continue to accumulate the organics to reduce the impacts of water deficit, in this period, the accumulated organics were used in the cell wall synthesis and other process related to the fruit development in order to make up for the loss of photosynthetic production decrease [57]. However, the long period of water deficit resulted in the elasticity loss of cell walls, thus led to the decreased yields. The salinity-induced yield reductions could result from decreased inflow of water into the fruits [58], and under saline water irrigation, the reduction in fruit yield corresponded to reductions in the fruit weight and number [59]. In this study, high salinity combined with low irrigation quota (S2W1) obtained the lowest tomato yield, which was in consistent with the early studies.

*BER* is commonly regarded as a calcium-deficient physiological disease [60], and water and salt are two improvement environmental factors affected its incidence. Selby [61] noted that the *BER*_*i*_ was related to the soil moisture, and further study demonstrated that the deficient or excessive water both increased the *BER*_*i*_. Mohamed [62] also found that the *BER*_*i*_ under excessive water supply was significantly higher than that under normality. In our study, the *BER*_*i*_ was negatively related to the irrigation quota, indicating that 360 mm irrigation quota was not excessive according to Mohamed’s study. However, some studies proved that the water affected the *BER*_*i*_ was an accidental phenomena [63–64]. In addition, our study also showed that the high salinity obviously increased the *BER*_*i*_ (of which S2W1 was the highest), it might because that the high salinity of irrigation water impeded the water absorption of tomato fruits [65], thus the calcium was difficult to move from the tomato root to the fruit bottom [66].

After one season tomato cultivation, the salt mass fraction of different soil layers that using saline water were increased to various degrees compared to that of freshwater, and the salts mainly accumulated in the plough layer. Yang [67] conducted a similar study as us but obtained a much higher increase of soil salt mass fraction in the plough layer, this may have occurred because that the temperature was relatively lower at the end of our experiment, thus the soil resalinization was slighter. Moreover, soil salts had a tendency to move to the deeper soil layer when using 320 mm and 360 mm irrigation quota, this agrees with Wang’s [68] study conclusions. In our study, the salt distribution might not be affected by only irrigation amount, but also by the mulching. Taia A. Abd El-Mageed [69] conducted a thorough experiment and pointed out that noticeable decrease in salts accumulation in the root zone could be associated with soil mulching application.

Projection pursuit model has been widely used in the optimization of irrigation regime. Hou [70] adopted the projection pursuit model to select the best drip irrigation scheme for crop. Other methods, such as principal component analysis, entropy weight coefficient model were also used for guiding the agricultural production [71]. The essence of these models was the same, namely realizing the reduction of high-dimensional data. When using the models to select an irrigation scheme, various indexes should be considered. However, most early studies noticed only the crop yield, water use and irrigation water consumption. Wu [72] took the crop growth, *Y*_*t*_, overall quality and water use into consideration when choosing a saline water irrigation regime, but has not involved the amount of residual salt in soil.

For northern China, a good scheme of saline water irrigation for tomatoes should not only consider the quality and *Y*_*t*_, but also involve the *Y*_*m*_, *IWUE* and the soil salt residual after irrigation. In this study, the optimal saline water irrigation scheme selected by the PP model was S1W3, 3.0 dS/m salinity combined with 360 mm irrigation quota. PP model avoided the one-sidedness of using the subjective weight independently, thus the results that were obtained were more reliable. The optimal treatment S1W3 in this study possessed the best comprehensive benefit (tomato overall quality, *Y*_*t*_, *Y*_*m*_, *IWUE* and soil salt residual), and was recommended as the saline water irrigation scheme for tomatoes in northern China. However, in this experiment, the irrigation waters were distributed evenly according to the whole growth stage of tomatoes, the uneven distribution of saline waters according to different growth stage of tomatoes may have different effects on the tomato growth and development. Therefore, more researches on this topic are needed in future.

## Conclusion

Compared to freshwater, saline water irrigations decreased the *LAI*_*m*_ of tomatoes, and *LAI*_*m*_ presented a decline tendency with higher salinity and lower irrigation quota. The *ρ*_*F*_, *D*_*s*_, *G*, *V*_*C*_ and *RSA* increased but the *V*_*F*_ decreased as the salinity increased and irrigation quota decreased. S2W1 treatment obtained the best overall quality of tomatoes, with the comprehensive quality index of 3.61. A higher salinity and lower irrigation quota caused a decrease in individual fruit weight and an increase in *BER*_*i*_, finally led to a reduction in *Y*_*t*_ and *Y*_*m*_. After one growth season of tomato, the mass fraction of soil salts in plough layer with S2W1 treatment was the highest, and which presented a decline trend with increasing irrigation quota. Moreover, compared to W1, soil salts had a tendency to move to the deeper soil layer when using W2 and W3 irrigation quota. According to the calculation results of PP model, S1W3 was the optimal treatment, which possessed the best comprehensive benefit (tomato overall quality, *Y*_*t*_, *Y*_*m*_, *IWUE* and soil salt residual), and was recommended as the saline water irrigation scheme for tomatoes in northern China.
